# Reference Values for the Rivermead Post-Concussion Symptoms Questionnaire (RPQ) from General Population Samples in the United Kingdom, Italy, and The Netherlands

**DOI:** 10.3390/jcm11164658

**Published:** 2022-08-09

**Authors:** Marina Zeldovich, Fabian Bockhop, Amra Covic, Katrin Cunitz, Suzanne Polinder, Juanita A. Haagsma, Nicole von Steinbuechel, on behalf of the CENTER-TBI Participants and Investigators

**Affiliations:** 1 Institute of Medical Psychology and Medical Sociology, University Medical Center Göttingen, Waldweg 37A, 37073 Göttingen, Germany; 2 Department of Public Health, Erasmus MC, University Medical Center Rotterdam, 3000 CA Rotterdam, The Netherlands

**Keywords:** traumatic brain injury, post-concussion symptoms, reference values, Rivermead Post-Concussion Symptoms Questionnaire, patient-reported outcome instruments

## Abstract

After traumatic brain injury (TBI), individuals may experience short- or long-term health burdens, often referred to as post-concussion symptoms (PCS). The Rivermead Post-Concussion Symptoms Questionnaire (RPQ) is one of the commonly used instruments to assess self-reported PCS. To date, no reference values for RPQ have been provided, although they are crucial for clinical practice when evaluating a patient’s health status relative to a comparable healthy population. Therefore, the aim of this study is to provide reference values for the United Kingdom, the Netherlands, and Italy. A total of 11,759 individuals (50.3% women) from representative general population samples participated in an online survey (4646 individuals from the UK, 3564 from the Netherlands, and 3549 from Italy). The factorial structure of the RPQ was examined using confirmatory factor analysis (CFA), and results from the general population samples were compared with those from respective TBI samples recruited within the international CENTER-TBI study using multigroup CFA. Reference values were stratified by sex, health status, age, and education using percentiles. The three-factorial model outperformed the one-factorial structure. The general population samples were largely comparable to the corresponding TBI samples, except for items such as dizziness, vision, and sensory sensitivity, which can be considered more TBI-specific. Because of the significant differences between the general population samples, we provided reference values for the total score and for the somatic, emotional, and cognitive scales for each country separately. The reference values provided can now be used in clinical practice and research. Future studies should obtain stratified reference values for other countries and languages to improve accuracy in the diagnosis and treatment of symptom burden after TBI.

## 1. Introduction

Traumatic brain injury (TBI) is defined as a change in brain functioning caused by an external force [1]. Those affected often suffer from life-long limitations [2] and experience a range of physical, emotional, and cognitive disabilities. The effects of TBI limit functioning [1] of the affected individuals, and often lead to substantial burden for caregivers, family members [3], and health care systems [2]. Even after mild traumatic brain injury (scores of 13–15 on the Glasgow Coma Scale; GCS [4]), which accounts for the majority of all TBI cases [2], individuals do not fully recover to their premorbid level of functioning and report being bothered by a range of health problems [5].

After sustaining a TBI, individuals may experience short-term or long-lasting physical (e.g., headaches), cognitive (e.g., difficulties concentrating), and emotional/behavioral (e.g., fatigue) [6] symptoms. These symptoms are often collectively referred to as post-concussion symptoms (PCS). PCS typically emerge after mild to moderate TBI [7], but also individuals after a severe TBI frequently report comparable short- or long-lasting deficits termed post-concussion-like (PC-like) symptoms [8,9].

Clinicians and researchers often rely on self-reporting of the concerned individuals using patient-reported outcome measures (PROMs) to screen and assess PCS. Among the most frequently used PROMs for self-reported PCS is the Rivermead Post-Concussion Symptoms Questionnaire (RPQ) [7]. The RPQ was suggested in the Common Data Elements (CDE) recommendations for assessing symptom burden following TBI in adults [10,11]. The questionnaire requires rating of the presence of 16 symptoms (headaches, dizziness, nausea and/or vomiting, noise sensitivity, sleep disturbance, fatigue, irritability, depression, frustration, forgetfulness and poor memory, poor concentration, slow thinking, blurred vision, light sensitivity, double vision, and restlessness) during the past 24 h before the assessment compared to the health condition before TBI. This questionnaire has been translated into a wide range of languages [12] and is broadly applied in PCS assessment [13]. However, despite its wide application, no reference values have yet been established for the RPQ.

Voormolen et al. (2019) [14] found a high prevalence of PC-like symptoms in the general population samples from the United Kingdom, The Netherlands, and Italy. Overall, 45.1% rated at least three symptoms at least as mild with fatigue showing the highest prevalence (49.9%) followed by sleep disturbance (42.4%). In addition, 17.5% reported at least three symptoms as being a moderate problem. In extension to this finding, providing reliable information on the clinical relevance of PCS reported by individuals after TBI is crucial. Patients’ current health status after TBI can be evaluated most accurately based on the comparison of health values of a representative general population sample with similar characteristics (e.g., same gender, age, or initial health status).

The comparison of RPQ scores of individuals’ after TBI with reference values obtained from the general population is of interest especially for clinicians, but also for researchers in the field of TBI. Since values may vary from country to country, the identification of problems in a single individual (i.e., individual health status) benefits from the comparison with country-specific values collected from the reference population (i.e., healthy general population sample).

Before providing reference values, the applicability of the RPQ in general population samples should be investigated by applying psychometric testing of the RPQ in the general population and testing for the equivalent assessment of the construct (i.e., PCS) in general and TBI populations. If results suggest that RPQ scores from both populations would be considered comparable, reference values can be established.

The aims of the present study are:

1. To investigate the applicability of the RPQ in general population samples from the United Kingdom (English sample), the Netherlands (Dutch sample), and Italy (Italian sample);
2. To provide reference values for these three countries in order to increase reliability and validity of outcome assessments, thereby strengthening the psychometric properties of the RPQ.

The comparison of an individual’s health status after TBI to values of a healthy reference population can provide guidance for both clinical decision makers and researchers.

## 2. Materials and Methods

### 2.1. General Population Samples

#### 2.1.1. Data Collection

**Figure 1:**
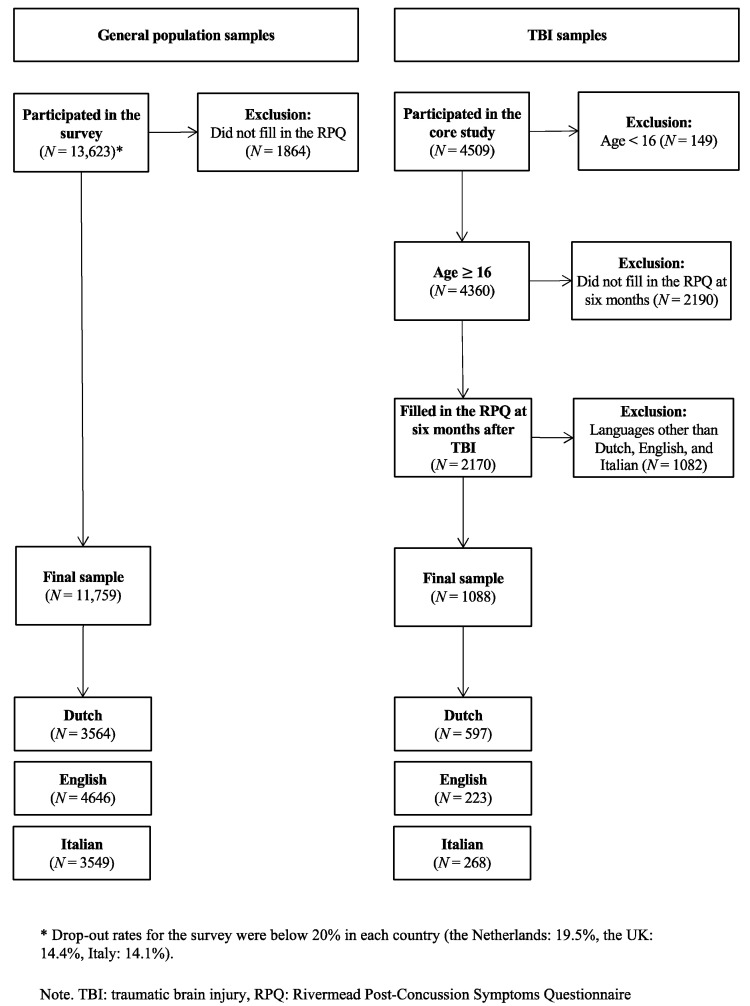
Composition of the study samples.

Data collection was carried out through an online survey from 29 June to 31 July 2017. Participant recruitment was handled by a private market research agency (https://www.dynata.com/, accessed on 20 June 2022), employed a large custom online panel and included survey dissemination, data processing, and hosting. The study sample was conceived to be representative with regard to the distribution of age, gender, and educational status in the United Kingdom, the Netherlands, and Italy, respectively. For more details, see Figure 1—left part “General population samples”.

To enhance representativeness by sampling participants from diverse social backgrounds, multiple recruitment sources were taken advantage of (e.g., proprietary loyalty partnerships, open recruitment to traditional online panels, and integrated partnerships with online communities, publishers, as well as social networks).

Study invitations did not include specific details on project aims to avoid self-selection bias, instead inviting suitable individuals to “take a survey”. Complete participation was reimbursed by the market research agency by handing out cash money, survey points, prizes, or sweepstakes. Participants who completed the survey in less than five minutes were classified as “speeders” and were deleted from the dataset. Respondents were required to answer every questionnaire item, since the electronic data collection system did not allow for missing responses. The recruitment process continued until the required quotas were met.

#### 2.1.2. Informed Consent

The recruiting agency obtained informed consent from all individuals who agreed to participate in the online survey. The process is described in the privacy agreement, which can be found at https://www.dynata.com/privacy-policy/ (accessed on 12 January 2022). Participants were informed on the survey welcome page as to the goal of the survey, which was to better understand the impact of TBI on patients’ lives, that the survey would take approximately 20 min to complete, as well as that all responses would be confidential and anonymous. The data were anonymized with each participant assigned a number in the order they completed the survey.

#### 2.1.3. Ethical Approval

The general population study was part of the Collaborative European NeuroTrauma Effectiveness Research (CENTER-TBI; clinicaltrials.gov NCT02210221) project. Ethical approval was obtained from the Leids Universitair Centrum—Commissie Medische Ethiek (approval P14.222/NV/nv).

### 2.2. TBI Samples

#### 2.2.1. Data Collection

Data collection for the TBI samples took place as part of the CENTER-TBI study between December 2014 and December 2019. A total of 4509 individuals after TBI from 63 centers in 18 European countries and Israel were enrolled in the core study. Inclusion criteria for the CENTER-TBI core study were the clinical diagnosis of TBI, indication for computed tomography (CT), admission within 24 h of injury, and informed consent for study participation. To avoid bias in outcome assessment, patients with severe preexisting neurologic disorders (e.g., epilepsy, stroke) were excluded from the study. Patients were either evaluated in the emergency room (ER) and then discharged or admitted to either the hospital ward or an intensive care unit (ICU). Further study details can be found elsewhere [15]. Data were retrieved from the CENTER-TBI database via the Neurobot tool (core data set 2.1, November 2019).

The following analyses comprised individuals belonging to the English, Dutch, and Italian language samples aged 16 years or older who filled out the RPQ six months after TBI (N = 1088). For more information, see Figure 1—right part “TBI samples”. Language samples composition has been described in more detail elsewhere [16].

#### 2.2.2. Informed Consent

Informed consent was obtained according to the respective local and national requirements for all patients recruited in the core dataset of CENTER-TBI and documented in the e-CRF [17].

#### 2.2.3. Ethical Approval

The CENTER-TBI study (EC grant 602150) has been conducted in accordance with all relevant laws of the EU if directly applicable or of direct effect and all relevant laws of the country where the recruiting sites were located, including but not limited to, the relevant privacy and data protection laws and regulations (the “Privacy Law”), the relevant laws and regulations on the use of human materials, and all relevant guidance relating to clinical studies from time to time in force including, but not limited to, the ICH Harmonized Tripartite Guideline for Good Clinical Practice (CPMP/ICH/135/95) (“ICH GCP”) and the World Medical Association Declaration of Helsinki entitled “Ethical Principles for Medical Research Involving Human Subjects”. Informed Consent was obtained for all patients recruited in the Core Dataset of CENTER-TBI and documented in the e-CRF. Ethical approval was obtained for each recruiting site. The list of sites, Ethical Committees, approval numbers, and approval dates can be found on the project’s website (https://www.center-tbi.eu/project/ethical-approval, accessed on 20 June 2022).

### 2.3. Sample Characteristics

All study participants provided information on their age, sex (TBI sample) or gender (general population samples), and level of education. Individuals from the general population samples additionally indicated whether they suffered from one or more chronic health conditions (i.e., asthma, heart disease, stroke, diabetes, back problems, osteoarthritis, rheumatism, cancer, memory problems due to a neurological condition such as dementia, memory problems due to aging, depression, or other problems). Information on chronic health conditions was merged for further analyses to test for differences regarding RPQ symptoms in individuals without chronic health conditions and participants suffering from at least one health problem. TBI severity has been assessed using the Glasgow Coma Scale (GCS) [4] with values 13–15 indicating mild, 9–12 indicating moderate, and less or equal to 8 indicating severe TBI.

### 2.4. The Rivermead Post-Concussion Symptoms Questionnaire

The Rivermead Post-Concussion Symptoms Questionnaire (RPQ) [7] is a self-assessment instrument comprising a list of 16 PCS. Respondents are asked to rate impairment associated with these symptoms during the past 24 h compared to their condition before TBI on a five-point Likert scale (from 0 “not experienced at all” to 4 “a severe problem”). Based on the originally proposed unidimensional factor structure, ratings are summarized into a total score which ranges from 0 to 64 with higher values indicating greater symptom severity.

The original English version of the RPQ alongside Dutch and Italian translations were used in this study. The latter versions were translated and linguistically validated in preparation for the CENTER-TBI study [12]. The RPQ was adapted for application in the general population by deleting any reference to TBI in the questionnaire introduction. Consequently, items such as “Compared with before the accident, do you now (i.e., over the last 24 h) suffer from…” were reworded to “Do you now (i.e., over the last 24 h) suffer from…”.

### 2.5. Statistical Analyses

#### 2.5.1. Descriptive Statistics and Psychometric Properties of the RPQ in General Population Samples

Investigation of the comparability of psychometric characteristics of the RPQ in general population samples with the results obtained from the TBI samples followed the approach described in the study by von Steinbuechel et al. (2021) [16]. Specifically, item characteristics per language version are provided including sample sizes, mean (*M*), standard deviation (*SD*), skewness (*SK*), and kurtosis (*KU*). *SK* and *KU* values from −2 to +2 were considered acceptable [18]. Response behavior was analyzed using absolute and relative frequencies in item categories.

Reliability analyses included Cronbach’s alpha (values from 0.70 to 0.95 indicating good to excellent internal consistency [19]), split-half reliability (odd vs. even items) with Spearman-Brown correction, and Cronbach’s alpha after omission of respective item (should not exceed the overall Cronbach’s alpha).

Item-total correlations were calculated to test the discriminatory quality of the items and correlation coefficients were taken as indicators of how well items discriminate between individuals with low as opposed to high symptom severity. Lower correlation coefficients indicate weaker discriminatory power and the cut-off was determined at a medium effect size (r ≥ 0.30) [20].

#### 2.5.2. Factorial Structure of the RPQ in General Population Samples

The factorial structure of the RPQ has been repeatedly discussed during the last decades. The initially proposed one-factor solution [7] has been more or less abandoned in favor of multi-factor solutions, of which several different models have been proposed [21,22,23,24,25,26,27]. However, there is still no consensus on the most suitable factorial structure.

One of the suggested RPQ structures is the three-factor solution reported in Smith-Seemiller and colleagues (2003) [21] covering *somatic* (nine items), *emotional* (four items), and *cognitive* (three items) scales. Evidence points to satisfactory fit of this model both in a cross-sectional investigation of six RPQ translations [28] as well as in a longitudinal study involving a large TBI sample [29]. Considering the satisfactory results in the TBI samples, we applied this factor solution to analyze factorial structure of the RPQ in general population groups in addition to the original single-factor model. Investigations utilized confirmatory factor analyses (CFA) with robust weighted least squares estimators (WLSMV) [30] for ordered categorical data in each language sample.

The goodness-of-fit of estimated models was evaluated with the help of multiple indices (cut-off criteria shown in brackets): $\chi^{2}$ and degrees of freedom (*df*), as well as the ratio $\chi^{2}/df$ (≤2) [31], the comparative fit index (CFI; ≥0.95) [32,33], the Tucker–Lewis index (TLI; ≥0.95) [33,34], the root mean square error of approximation (RMSEA; excellent fit at 0.05, mediocre fit at 0.10) [35,36] including 90% confidence interval (CI_90%_), and the standardized root mean square residual (SRMR; <0.08) [33]. All fit indices were considered simultaneously to evaluate the model fit since the indices have not yet been validated for ordinal data [37].

#### 2.5.3. Comparability of the RPQ Scores in General Population and TBI Samples

The comparability of the RPQ scores in general population and TBI samples was investigated for each language sample using a multi-group CFA approach (i.e., general population sample vs. TBI sample in each language), also referred to as measurement invariance (MI) testing. For this purpose, we followed the framework originally proposed by Wu and Estabrook (2016) [38] and updated by Svetina, Rutkowski, and Rutkowski (2020) [39]. First, we fitted the baseline model (1). Second, this model was then restrained by requiring measurement invariance of thresholds (2) and intercepts as well as thresholds (3) across the groups. Finally, differences in model fit were evaluated using chi-square difference test and changes in the comparative fit index (ΔCFI) and RMSEA (ΔRMSEA). Should the chi-square difference test not suggest significant differences, as well as ΔCFI < 0.01 [40], and ΔRMSEA ≤ 0.01 [41], models were considered equivalent. In that case, the simpler model with fewer restraints would be retained. The assumption of MI was considered reasonable when the baseline model was chosen over the restrained models. The RPQ scores in general population and TBI samples can be thus considered comparable allowing for calculation of the reference values derived from general population samples.

#### 2.5.4. Reference Values

To identify factors associated with PC-like symptoms in general population samples from the United Kingdom, Italy, and the Netherlands, linear regression models were estimated for the RPQ total score, and emotional, somatic, and cognitive scales, respectively. Country, sex, age, education, and health status as well as all possible second-order interactions between the factors (e.g., country × sex, country × age, country × health status etc.) served as independent variables. For RPQ scores, responses rated as 1 (no more of a problem than before) were recoded to 0 as proposed for the original scoring of the RPQ [7]. Significant factors were used to stratify reference values.

Reference values were computed based on percentiles which represent the value that a certain percentage of observations falls below. This information can be used to determine whether an individual’s RPQ value after TBI is below, equal to, or above the reference population value. For patient-level interpretation, the following percentiles are provided: 2.5%, 5%, 16%, 30%, 40%, 50%, 60%, 70%, 85%, 95%, and 97.5%. RPQ values that exceed the reference average by one standard deviation or more are considered clinically relevant [42]. This corresponds to the 85%-quantile in data that present a normal distribution. Interpretation examples are provided in the results section.

All analyses were carried out with R version 4.0.2. [43] and packages table1 [44] for descriptive analyses, psych [45] for psychometric properties, and lavaan [46] for the CFA and MI testing. The significance level was set at 5%.

## 3. Results

### 3.1. Sample Characteristics

Overall, 11,759 individuals (50.3% females) aged on average 44.6 ± 15.3 years (median: 44.0; range 18.0–75.0) from general population samples completed the questionnaire, including 4646 individuals from the United Kingdom, 3564 from the Netherlands, and 3549 from Italy. Most participants held a middle school degree (over 40% across all language samples). Approximately half of all participants (50.9%) reported to suffer from at least one chronic health condition. The total average RPQ score in all countries was 12.0 (SD = 13, median = 8), indicating clinically relevant symptom burden according to the mean but not the median when applying a cut-off of 12 [23].

The TBI sample consisted of 1088 individuals after TBI (35.6% females) aged on average 51.3 ± 19.2 years (median: 54.0; range: 16.0–95.0) from the Dutch (N = 597), English (N = 223), and Italian (N = 268) language samples. Most participants completed secondary or high school (52.1%) and sustained a mild TBI (74.3%). Average RPQ total score across the language groups was 14.7 (SD = 12.6, Median = 12), indicating clinically relevant impairment [23]. Individuals after mild TBI reported lower symptom severity on average (M = 14.1, SD = 12.6, Median = 12) compared with those who had sustained moderate or severe TBI (M = 16.2, SD = 12.4, Median = 13).

Comparison of the sample characteristics between the general population samples with respective TBI language samples revealed significant differences in distribution of sex and education (*p* < 0.001). Participants from the TBI samples were significantly older compared with those from the respective general population (*p* < 0.001; approx. five years on average). For more details, see Table 1 (left part—“*General population samples*”, right part—“*TBI samples*”).

Distribution of chronic health complaints reported by participants from general population samples is provided in the Table 2. Across all countries, the most commonly reported health complaints were depression (18.7%), other (13%), asthma (10.2%), and back pain (9.7%). The “other” category included an open text field for participants to complete. Multiple mentions of other health complaints included COPD, mental disorders other than depression, epilepsy, fibromyalgia, kidney disease, and others.

### 3.2. Item Characteristics and Psychometric Properties of the RPQ in the General Population Samples

Dizziness (22%), noise sensitivity (19.6%), light sensitivity (17.5%), nausea (14.3%), and blurred (18%) and double vision (9.4%) showed the lowest percentage of endorsement. In contrast, fatigue (41.4%), headaches (35.8%), sleeping problems (35.3%), and being irritable (34.3%) were more common among general population samples. For more detail, see Appendix A—Table A1.

On average, the distribution of RPQ items was less skewed across the general population samples (*SK: M* = 1.10, *SD* = 0.56, *KU: M* = 0.46, *SD* = 1.79) compared to the TBI samples (*SK*: *M* = 1.22, *SD* = 0.73; *KU*: *M* = 0.99, *SD* = 2.78) [16]. See Appendix A—Table A2 for more detail.

The RPQ showed excellent reliability in the general population samples. Cronbach’s alpha values were above 0.90 across the general population samples (Dutch: 0.94; English: 0.95; Italian: 0.92) and comparable with the TBI samples (Dutch: 0.93; English: 0.92; Italian: 0.91) [16]. The values of the Cronbach’s alpha if an item was omitted were smaller than the Cronbach’s alpha in each language sample. The item-total correlations were above 0.30 across all samples. For more detail, see Appendix A—Table A3.

### 3.3. Factorial Structure of the RPQ in the General Population Samples

Comparable to the results obtained from the respective TBI samples [16], the original one-factor structure could not be replicated for any of the general population samples. In contrast, the three-factor solution comprising *somatic*, *emotional*, and *cognitive* scales showed widely satisfactory results in all language samples (see Appendix A—Table A4). Consequently, the following comparisons of general population samples with TBI samples were based on this factorial solution.

### 3.4. Measurement Invariance

**Figure A2:**
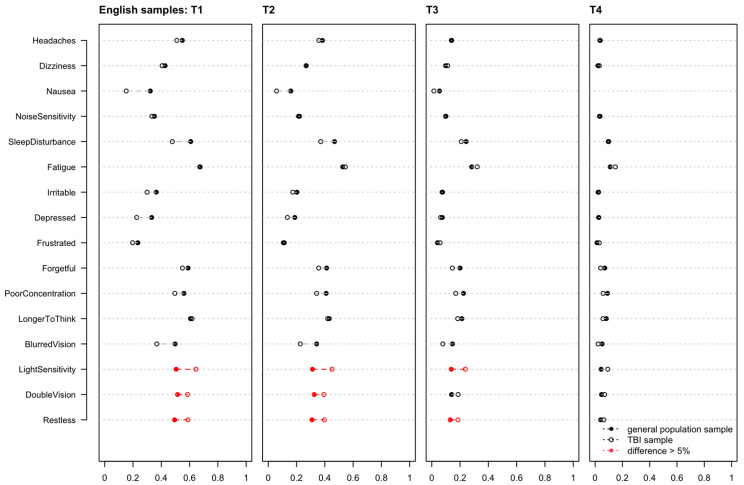
Relative difference between thresholds models (inverse of the back-transformed thresholds) of the English general population and TBI samples. Items marked with red color indicate difference above acceptable cut-off of 5%.

**Figure A3:**
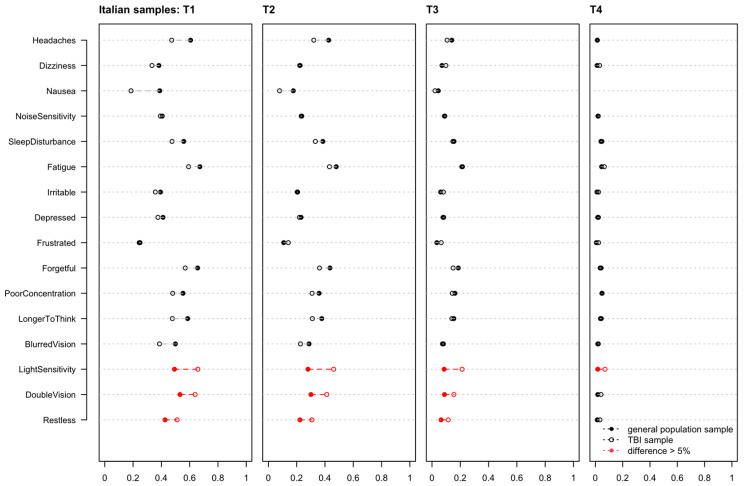
Relative difference between thresholds models (inverse of the back-transformed thresholds) of the Italian general population and TBI samples. Items marked with red color indicate difference above acceptable cut-off of 5%.

The MI analyses between general population samples and TBI samples revealed non-significant differences between the baseline models (1) and the thresholds models (2) in the English language sample and significant differences in Dutch and Italian samples. There were no differences between threshold models (2) and intercepts and threshold models (3) across all language samples (see Appendix A—Table A5, *upper part*). Significant differences between the baseline (1) and threshold models (2) in the Dutch and Italian samples indicated differences in the probability of choosing response categories between the general population samples and the TBI samples. The ΔCFI and ΔRMSEA values did not exceed the cut-off values, and the relative differences between the samples in most of the items were negligible (i.e., they did not exceed 5%). However, some items exceed the 5%-cut-off across all language samples (i.e., light sensitivity, double vision, and restlessness in all language samples and dizziness, noise sensitivity, and fatigue in Dutch samples). For further details, see Appendix A—Figure A1, Figure A2 and Figure A3.

In addition, the comparison between the general population samples (i.e., Dutch vs. English vs. Italian) showed significant differences across constrained models (see Appendix A—Table A5, *lower part*).

Given that most of the differences in symptoms between the language samples did not exceed the permissible cut-off value, that most of the symptoms characterized by the significant differences were specifically relevant to TBI and rarely occur in the healthy general population [47,48], and that thresholds and intercepts and threshold models did not show significant differences in all samples, it seemed appropriate to establish reference values using the data obtained from the general population samples. Furthermore, the violation of the MI assumption between general population samples at all levels suggested that providing separate reference values for each language sample is reasonable.

### 3.5. Regression Analyses

The results of the regression analyses indicated significant effects of all independent variables on both the RPQ total score and the scales, suggesting the provision of separate reference scores for countries stratified by sociodemographic and health factors. However, considering the interactions between factors, education was found to have a significant relationship with RPQ scores only in combination with country. Therefore, reference values for each country were reported stratified by sex, age, and health status information. For more details on the results of the regression analyses, see Appendix A—Table A6.

### 3.6. Reference Values

Reference values for the RPQ total score obtained from the English, Italian, and Dutch general samples are presented in Table 3, Table 4 and Table 5. Table A7, Table A8 and Table A9 in Appendix B provide reference values for the scales. Below we provide an example for application of the reference values.

A 45-year-old healthy woman from the UK had sustained a TBI. Her RPQ total score was 25. Table 2 shows that 95% of healthy individuals in her respective age, gender, and health status group report the same or lower symptom severity. In other words: Only 5% of the reference population suffer from more severe symptoms. Therefore, the reported RPQ symptoms are rated as clinically relevant.

If this woman additionally reports at least one chronic health condition (e.g., asthma prior TBI), approximately 60% of the reference population from her age, gender, and health status group report less severe symptoms and 40% of the general population with similar characteristics report more severe PCS than she does. In this case, the woman’s individual score is within the normal range.

## 4. Discussion

The present study aimed to supply clinicians and researchers with reference values for the RPQ obtained from general population samples from the United Kingdom, Italy, and the Netherlands. These samples were designed to be representative for the general population in the respective countries and thus provide a reliable basis for reference values.

The results showed that the factorial structure of the RPQ based on the general population data reflects the problems reported in the TBI population [21,22,23,24,25,26,27]. This underlines problems with replicability of the original one-factor solution. Therefore, in addition to the reference values based on the original RPQ total score, we derived reference values for the three scales covering somatic, emotional, and cognitive symptoms which showed a good fit in previous studies [28,29]. As there is no consensus yet on the underlying factorial structure of the RPQ, we found this additional information helpful to clinicians in assessing each patient’s PCS.

Differences between the language samples indicate the need for language- and country-specific reference values. These findings can be supported by previous research, although relatively little has been published on PC-like symptoms in general population samples or direct comparisons between healthy individuals from different countries. Voormolen et al. [14] documented the highest reported number of PC-like symptoms in the English general population sample (47.8%) closely followed by the Italian sample (46.2%), when using a cut-off of 2 implying rating single RPQ symptoms as at least mild. Applying a cut-off of 3 (i.e., reporting at least a moderate problem), the participants from the United Kingdom again exhibited most symptoms (20.9%), followed by individuals from the Dutch sample (16.3%). In other English-speaking countries, participants from general population samples report varying levels of PC-like symptoms. A Canadian study [49] found that 35.9% to 71.8% of healthy community volunteers experienced one or more of the 13 symptoms—comparable to those assessed with the RPQ–of the British Columbia Post-Concussion Symptom Inventory (BC-PSI) [50] at least once or twice in the past two weeks. Overall, between 24.1% (noise sensitivity) and 57.3% (fatigue and feeling nervous) of symptoms were rated as at least mild by participants in an Australian student and community sample using the BC-PSI [51].

Given the variability between the general populations of different countries in the prevalence of PC-like symptoms, we would encourage the use of language/country-specific reference values rather than relying on the commonly proposed cut-off values (e.g., a cut-off of 12 proposed by Potter et al. [23]).

Apart from country-specific findings, we generally found a relatively high prevalence of PC-like symptoms in the general population samples included in the present study. This is largely consistent with previous research findings. In recent decades, the rate of self-reported symptoms in healthy individuals has been considered high to very high [14,47,48,49,51,52], indicating the need to account for this when diagnosing PCS in individuals after TBI. In addition, as reflected by the distribution of reference values, these symptoms are more prevalent in the general population subsamples with chronic health complaints. Some studies have shown that the occurrence of PCS is less TBI-specific [53,54] and more related to the premorbid health status of the affected individuals than to TBI [55,56]. Furthermore, individuals suffering from chronic health conditions may experience cognitive, emotional, and somatic symptoms comparable to PCS (e.g., chronic pains [21], depression [51], stroke and multiple sclerosis [57], etc.). However, this does not mean that symptoms that appear to be due to conditions other than TBI should be ignored or left untreated. Rather, they indicate that patients may have multiple needs that may be interacting and hindering the healing process. When possible, further differential diagnosis should be made to provide the best possible treatment for the affected individual.

Considering this information, we strongly recommend assessing the premorbid health status of TBI patients or individuals with suspected TBI to determine the most appropriate reference population for use of the RPQ. Only by comparing individuals after TBI with a reasonably similar reference population, results can be reliably classified, and appropriate measures and targeted therapeutic interventions derived. Since the selection of reference values is crucial for the assessment of the individual symptomatology as well as the derived treatment and its success, in the clinical context, an accurate collection of medical history is necessary to select the appropriate reference population. With the reference values presented here, clinicians have now the opportunity to consider the most important factors (i.e., gender, age, education, and health status) when assessing patient outcomes after TBI with the RPQ.

### Strengths and Limitations

This study has important strengths as well as some limitations. Due to the large sample size and the representativeness of the quotas for gender, age, and education level in the language samples, we were able to provide reliable reference values for further application in clinical practice and research for the United Kingdom, Italy, and the Netherlands. In addition, we considered health status in establishing reference values, which allows for a more detailed evaluation of PCS or PC-like symptoms in single individuals after TBI.

However, some issues related to the nature of data collection should be considered. First, participants were recruited exclusively through Internet platforms via online surveys which are often associated with (self-)selection and response bias [58]. Furthermore, we do not have information on the number of individuals contacted who declined to participate in the study as well as no characteristics of those who dropped out during the survey. Second, we lacked information on a possible TBI experience in the general population samples. Therefore, despite all attempts made during data collection, some sample selection bias may occur (e.g., only individuals with access to the Internet were able to complete the panel). These points as well as the legitimacy of data use, have been discussed in more detail elsewhere [59]. Finally, because of the relatively small number of cases within specific groups of chronic conditions, further stratification of reference values was not possible. Therefore, distinguishing between minor and severe chronic health conditions is not possible when applying reference values presented here. Since the effects of chronic health conditions may influence symptom burden in different ways, future studies should focus on differences in PC-like symptoms in specific diseases.

Furthermore, the relatively small and unequally distributed across the countries sample size of the TBI language samples used in the MI analyses could have had an impact on the results since there was a low variability in responses and extreme answer categories of some items were rarely endorsed. Finally, characteristics between general population and TBI samples differed significantly which could have impacted the results.

## 5. Conclusions

This study provides RPQ reference values for three European countries that are ready for use in clinical practice and research. Future studies should target reference values for other countries and languages to improve accuracy in the diagnosis and treatment of PCS after TBI.

## Figures and Tables

**Table 1:** Sample characteristics.

		General Population Samples				TBI Sample				Comparison ^5^
	Group	English	Italian	Dutch	Total	English	Dutch	Italian	Total	*p*
Variable	(General Population Samples/ *TBI Samples*)	(N = 4646)	(N = 3549)	(N = 3564)	(N = 11759)	(N = 223)	(N = 597)	(N = 268)	(N = 1088)	
Age	Mean (SD)	44.1 (15.6)	45.0 (14.8)	44.8 (15.3)	44.6 (15.3)	48.3 (17.1)	52.9 (19.1)	50.2 (20.6)	51.3 (19.2)	All group comparisons < 0.001
	Median [Min, Max]	44.0 [18.0, 75.0]	45.0 [18.0, 75.0]	45.0 [18.0, 75.0]	44.0 [18.0, 75.0]	51.0 [16.0, 85.0]	57.0 [16.0, 95.0]	53.0 [16.0, 93.0]	54.0 [16.0, 95.0]	
Age in groups ^1^	18–24/*16–24*	601 (12.9%)	346 (9.7%)	429 (12.0%)	1376 (11.7%)	29 (13.0%)	74 (12.4%)	46 (17.2%)	149 (13.7%)	-
	25–34	880 (18.9%)	608 (17.1%)	598 (16.8%)	2086 (17.7%)	25 (11.2%)	57 (9.5%)	30 (11.2%)	112 (10.3%)	
	35–44	918 (19.8%)	806 (22.7%)	732 (20.5%)	2456 (20.9%)	28 (12.6%)	56 (9.4%)	29 (10.8%)	113 (10.4%)	
	45–54	912 (19.6%)	726 (20.5%)	743 (20.8%)	2381 (20.2%)	60 (26.9%)	84 (14.1%)	39 (14.6%)	183 (16.8%)	
	55–64	765 (16.5%)	612 (17.2%)	646 (18.1%)	2023 (17.2%)	44 (19.7%)	142 (23.8%)	48 (17.9%)	234 (21.5%)	
	65–75/*65+*	570 (12.3%)	451 (12.7%)	416 (11.7%)	1437 (12.2%)	37 (16.6%)	184 (30.8%)	76 (28.4%)	297 (27.3%)	
Gender/*sex* ^2^	Male	2288 (49.2%)	1770 (49.9%)	1782 (50.0%)	5840 (49.7%)	74 (33.2%)	229 (38.4%)	84 (31.3%)	387 (35.6%)	All group comparisons < 0.001
	Female	2358 (50.8%)	1779 (50.1%)	1782 (50.0%)	5919 (50.3%)	149 (66.8%)	368 (61.6%)	184 (68.7%)	701 (64.4%)	
Education ^3^	Low/ *primary school/none/unknown*	1066 (22.9%)	1200 (33.8%)	1064 (29.9%)	3330 (28.3%)	2 (0.9%)	40 (6.7%)	64 (23.9%)	106 (9.7%)	All group comparisons < 0.001
	Middle/ *secondary school/high school*	1986 (42.7%)	1968 (55.5%)	1601 (44.9%)	5555 (47.2%)	140 (62.8%)	363 (60.8%)	64 (23.9%)	567 (52.1%)	
	High/ *post-high school*	1594 (34.3%)	381 (10.7%)	899 (25.2%)	2874 (24.4%)	61 (27.4%)	125 (20.9%)	101 (37.7%)	287 (26.4%)	
	Missing	-	-	-	-	20 (9.0%)	69 (11.6%)	39 (14.6%)	128 (11.8%)	
Chronic health complaints/ *TBI severity* ^4^	no chronic health complaints/ *mild TBI*	2159 (46.5%)	1940 (54.7%)	1677 (47.1%)	5776 (49.1%)	160 (71.7%)	474 (79.4%)	174 (64.9%)	808 (74.3%)	
	at least one chronic health complaint/ *moderate/severe TBI*	2487 (53.5%)	1609 (45.3%)	1887 (52.9%)	5983 (50.9%)	62 (27.8%)	108 (18.1%)	94 (35.1%)	264 (24.3%)	-
	Missing	-	-	-	-	1 (0.4%)	15 (2.5%)	0 (0%)	16 (1.5%)	
RPQ total score	Mean (SD)	13.2 (14.0)	11.6 (11.9)	10.7 (12.4)	12.0 (13.0)	15.1 (12.7)	14.8 (13.0)	14.1 (11.7)	14.7 (12.6)	-
	Median [Min, Max]	8.00 [0, 64.0]	8.00 [0, 64.0]	6.00 [0, 64.0]	8.00 [0, 64.0]	13.0 [0, 56.0]	12.0 [0, 64.0]	12.0 [0, 56.0]	12.0 [0, 64.0]	-
Somatic scale	Mean (SD)	6.52 (7.14)	6.03 (6.37)	5.71 (6.64)	6.13 (6.77)	7.02 (6.41)	7.15 (6.72)	6.75 (6.03)	7.02 (6.49)	-
	Median [Min, Max]	4.00 [0, 36.0]	4.00 [0, 36.0]	4.00 [0, 36.0]	4.00 [0, 36.0]	6.00 [0, 30.0]	5.00 [0, 36.0]	6.00 [0, 29.0]	5.50 [0, 36.0]	-
Emotional scale	Mean (SD)	4.27 (4.79)	3.66 (4.20)	2.97 (4.08)	3.69 (4.44)	4.12 (4.00)	3.76 (4.14)	3.78 (3.78)	3.84 (4.03)	-
	Median [Min, Max]	2.00 [0, 16.0]	2.00 [0, 16.0]	0 [0, 16.0]	2.00 [0, 16.0]	3.00 [0, 16.0]	3.00 [0, 16.0]	3.00 [0, 16.0]	3.00 [0, 16.0]	-
Cognitive Scale	Mean (SD)	2.43 (3.47)	1.90 (2.90)	2.05 (3.14)	2.15 (3.22)	3.92 (3.65)	3.85 (3.51)	3.62 (3.24)	3.81 (3.47)	-
	Median [Min, Max]	0 [0, 12.0]	0 [0, 12.0]	0 [0, 12.0]	0 [0, 12.0]	3.00 [0, 12.0]	3.00 [0, 12.0]	3.00 [0, 12.0]	3.00 [0, 12.0]	-

^1^ Age in groups slightly differs between general population samples (i.e., 18–24 and 65–75) and TBI samples (i.e., 16–24, 65+). ^2^ Gender is provided for the general population samples, information on sex is collected for the TBI samples. ^3^ Educational attainment was categorized as low (lower school), middle (comprehensive school), and high (college and university) for the general population samples and primary school, secondary/high school and post-high school for the TBI samples. ^4^ Chronic health complaints are provided for the general population samples, information on TBI severity (GCS; mild ≥ 13, moderate/severe ≤ 12) is reported for the TBI samples. ^5^ General population and TBI samples were compared in terms of age in years (*t*-test), sex, and education (Chi-squared-test); significance level was set at 5%. Note. N: absolute frequencies; %: relative frequencies; SD: standard deviation; Min: minimum; Max: maximum.

**Table 2:** Distribution of chronic health complaints reported by participants from general population samples.

	English	Italian	Dutch	Total
	**N (%)**	**N (%)**	**N (%)**	**N (%)**
Asthma	602 (13.0%)	258 (7.3%)	336 (9.4%)	1196 (10.2%)
Heart Disease	109 (2.3%)	68 (1.9%)	102 (2.9%)	279 (2.4%)
Stroke	74 (1.6%)	40 (1.1%)	81 (2.3%)	195 (1.7%)
Diabetes	390 (8.4%)	247 (7.0%)	274 (7.7%)	911 (7.7%)
Back complaints	567 (12.2%)	224 (6.3%)	355 (10.0%)	1146 (9.7%)
Arthrosis	141 (3.0%)	345 (9.7%)	346 (9.7%)	832 (7.1%)
Rheumatism	192 (4.1%)	235 (6.6%)	218 (6.1%)	645 (5.5%)
Cancer	128 (2.8%)	66 (1.9%)	140 (3.9%)	334 (2.8%)
Dementia	82 (1.8%)	62 (1.7%)	94 (2.6%)	238 (2.0%)
Aging problems	205 (4.4%)	149 (4.2%)	82 (2.3%)	436 (3.7%)
Depression	1254 (27.0%)	522 (14.7%)	423 (11.9%)	2199 (18.7%)
Other ^1^	493 (10.6%)	354 (10.0%)	687 (19.3%)	1534 (13.0%)
Total	4646 (100%)	3549 (100%)	3564 (100%)	11,759 (100%)

^1^ Choosing category “other” implied adding a comment in an open text field. Note. N = absolute frequencies, relative frequencies.

**Table 3:** Reference values for the RPQ total score obtained from the English general population sample stratified by sex, health status, and age.

Gender × Health Status × Age				Low Symptoms Severity		−1 SD			Md			+1 SD		High Symptoms Severity
Gender	Health status	Age	N	2.5%	5%	16%	30%	40%	50%	60%	70%	85%	95%	97.5%
Male	Healthy	18–40	566	0	0	0	0	0	2	5	8	16	30	33
		41–64	460	0	0	0	0	0	0	2	4	10	21	28
		65–75	118	0	0	0	0	0	0	2	4	9	21	24
	At least one chronic health condition	18–40	446	0	0	4	9	15	19	24	28	36	46	50
		41–64	542	0	0	2	6	10	15	19	24	34	48	53
		65–75	156	0	0	0	2	4	6	11	13	24	31	40
Female	Healthy	18–40	474	0	0	0	0	3	6	8	12	21	31	34
		41–64	422	0	0	0	0	0	3	4	8	16	25	30
		65–75	119	0	0	0	0	0	2	3	4	10	18	24
	At least one chronic health condition	18–40	564	0	0	7	13	17	21	25	29	37	49	52
		41–64	602	0	0	6	10	15	20	24	29	38	48	52
		65–75	177	0	0	2	4	7	10	12	16	25	36	39
		Total	4646	0	0	0	2	5	8	13	19	30	41	48

Note: 50% percentiles represent 50% of the distribution corresponding to the median (Md); SD: standard deviation; values from −1 standard deviation (16%) to +1 standard deviation (85%) are within the normal range (i.e., not clinically relevant symptom intensity); values below 16% indicate low symptoms intensity (i.e., absence of RPQ symptoms) and values above 85% indicate high symptom intensity (i.e., presence of clinically relevant RPQ symptoms).

**Table 4:** Reference values for the RPQ total score obtained from the Italian general population sample stratified by sex, health status, and age.

Gender × Health Status × Age				Low Symptoms Severity		−1 SD			Md			+1 SD		High Symptoms Severity
Gender	Health status	Age	N	2.5%	5%	16%	30%	40%	50%	60%	70%	85%	95%	97.5%
Male	Healthy	18–40	467	0	0	0	0	2	3	4	7	14	26	32
		41–64	454	0	0	0	0	0	2	4	7	14	26	32
		65–75	106	0	0	0	0	0	2	4	6	10	17	19
	At least one chronic health condition	18–40	227	0	0	2	7	11	15	18	23	31	38	39
		41–64	391	0	0	2	4	8	11	15	20	29	39	44
		65–75	125	0	0	0	2	5	8	10	14	19	31	36
Female	Healthy	18–40	419	0	0	0	3	4	7	11	15	21	30	32
		41–64	403	0	0	0	2	4	6	8	12	20	31	33
		65–75	91	0	0	0	0	0	2	6	7	12	24	31
	At least one chronic health condition	18–40	302	0	0	5	11	15	17	22	27	33	40	44
		41–64	435	0	0	6	10	14	17	20	24	33	42	47
		65–75	129	0	0	2	7	10	12	16	18	27	37	39
		Total	3549	0	0	0	2	5	8	12	16	25	35	40

Note: 50% percentiles represent 50% of the distribution corresponding to the median (Md); SD: standard deviation; values from −1 standard deviation (16%) to +1 standard deviation (85%) are within the normal range (i.e., not clinically relevant symptom intensity); values below 16% indicate low symptoms intensity (i.e., absence of RPQ symptoms) and values above 85% indicate high symptom intensity (i.e., presence of clinically relevant RPQ symptoms).

**Table 5:** Reference values for the RPQ total score obtained from the Dutch general population sample stratified by sex, health status, age, and education level.

Gender × Health Status × Age				Low Symptoms Severity		−1 SD			Md			+1 SD		High Symptoms Severity
Gender	Health status	Age	N	2.5%	5%	16%	30%	40%	50%	60%	70%	85%	95%	97.5%
Male	Healthy	18–40	444	0	0	0	0	0	2	4	6	14	27	32
		41–64	412	0	0	0	0	0	0	2	4	11	21	28
		65–75	94	0	0	0	0	0	0	0	2	4	8	10
	At least one chronic health condition	18–40	276	0	0	0	6	8	14	19	24	33	44	49
		41–64	447	0	0	0	4	7	10	15	20	29	42	47
		65–75	109	0	0	0	0	2	4	6	10	18	28	35
Female	Healthy	18–40	363	0	0	0	0	2	4	6	10	18	28	32
		41–64	298	0	0	0	0	2	2	4	8	13	23	29
		65–75	66	0	0	0	0	0	2	3	4	10	18	19
	At least one chronic health condition	18–40	372	0	0	5	11	14	18	21	26	33	45	51
		41–64	536	0	0	2	6	8	12	16	21	29	37	44
		65–75	147	0	0	0	4	6	8	11	15	22	32	35
		Total	3564	0	0	0	2	4	6	9	14	25	35	43

Note: 50% percentiles represent 50% of the distribution corresponding to the median (Md); SD: standard deviation; values from −1 standard deviation (16%) to +1 standard deviation (85%) are within the normal range (i.e., not clinically relevant symptom intensity); values below 16% indicate low symptoms intensity (i.e., absence of RPQ symptoms) and values above 85% indicate high symptom intensity (i.e., presence of clinically relevant RPQ symptoms).

**Figure A1:**
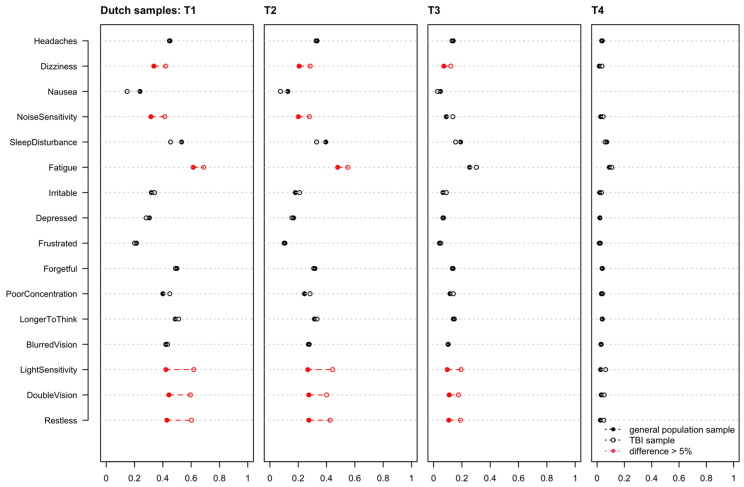
Relative difference between thresholds models (inverse of the back-transformed thresholds) of the Dutch general population and TBI samples. Items marked with red color indicate difference above acceptable cut-off of 5%.

## Data Availability

All relevant data are available upon request from CENTER-TBI, and the authors are not legally allowed to share it publicly. The authors confirm that they received no special access privileges to the data. CENTER-TBI is committed to data sharing and in particular to responsible further use of the data. Hereto, we have a data sharing statement in place: https://www.center-tbi.eu/data/sharing. The CENTER-TBI Management Committee, in collaboration with the General Assembly, established the Data Sharing policy, and Publication and Authorship Guidelines to assure correct and appropriate use of the data as the dataset is hugely complex and requires help of experts from the Data Curation Team or Bio- Statistical Team for correct use. This means that we encourage researchers to contact the CENTER-TBI team for any research plans and the Data Curation Team for any help in appropriate use of the data, including sharing of scripts. Requests for data access can be submitted online: https://www.center-tbi.eu/data (accessed on 12 January 2022). The complete Manual for data access is also available online: https://www.center-tbi.eu/files/SOP-Manual-DAPR-2402020.pdf (accessed on 12 January 2022).

## Supplementary Materials

The following supporting information can be downloaded at: https://www.mdpi.com/article/10.3390/jcm11164658/s1.

## Appendix A

**Table A1:** Distribution of the item responses of the RPQ in the general population samples.

Item	Response Category	English	Italian	Dutch	Total
		(N = 4646)	(N = 3549)	(N = 3564)	(N = 11,759)
Headaches	0	2094 (45.1%)	1399 (39.4%)	1963 (55.1%)	5456 (46.4%)
	1	764 (16.4%)	625 (17.6%)	381 (10.7%)	1770 (15.1%)
	2	1137 (24.5%)	1033 (29.1%)	736 (20.7%)	2906 (24.7%)
	3	499 (10.7%)	442 (12.5%)	360 (10.1%)	1301 (11.1%)
	4	152 (3.3%)	50 (1.4%)	124 (3.5%)	326 (2.8%)
Feeling of Dizziness	0	2661 (57.3%)	2200 (62.0%)	2374 (66.6%)	7235 (61.5%)
	1	741 (15.9%)	553 (15.6%)	445 (12.5%)	1739 (14.8%)
	2	792 (17.0%)	540 (15.2%)	487 (13.7%)	1819 (15.5%)
	3	356 (7.7%)	211 (5.9%)	203 (5.7%)	770 (6.5%)
	4	96 (2.1%)	45 (1.3%)	55 (1.5%)	196 (1.7%)
Nausea and/or Vomiting	0	3145 (67.7%)	2174 (61.3%)	2715 (76.2%)	8034 (68.3%)
	1	766 (16.5%)	746 (21.0%)	397 (11.1%)	1909 (16.2%)
	2	481 (10.4%)	470 (13.2%)	278 (7.8%)	1229 (10.5%)
	3	176 (3.8%)	137 (3.9%)	131 (3.7%)	444 (3.8%)
	4	78 (1.7%)	22 (0.6%)	43 (1.2%)	143 (1.2%)
Noise Sensitivity, easily upset by loud noise	0	3012 (64.8%)	2114 (59.6%)	2450 (68.7%)	7576 (64.4%)
	1	611 (13.2%)	587 (16.5%)	387 (10.9%)	1585 (13.5%)
	2	569 (12.2%)	536 (15.1%)	398 (11.2%)	1503 (12.8%)
	3	308 (6.6%)	247 (7.0%)	243 (6.8%)	798 (6.8%)
	4	146 (3.1%)	65 (1.8%)	86 (2.4%)	297 (2.5%)
Sleep Disturbance	0	1830 (39.4%)	1582 (44.6%)	1682 (47.2%)	5094 (43.3%)
	1	634 (13.6%)	585 (16.5%)	455 (12.8%)	1674 (14.2%)
	2	1050 (22.6%)	828 (23.3%)	753 (21.1%)	2631 (22.4%)
	3	686 (14.8%)	412 (11.6%)	422 (11.8%)	1520 (12.9%)
	4	446 (9.6%)	142 (4.0%)	252 (7.1%)	840 (7.1%)
Fatigue, tiring more easily	0	1529 (32.9%)	1176 (33.1%)	1392 (39.1%)	4097 (34.8%)
	1	674 (14.5%)	665 (18.7%)	450 (12.6%)	1789 (15.2%)
	2	1132 (24.4%)	953 (26.9%)	818 (23.0%)	2903 (24.7%)
	3	792 (17.0%)	594 (16.7%)	581 (16.3%)	1967 (16.7%)
	4	519 (11.2%)	161 (4.5%)	323 (9.1%)	1003 (8.5%)
Being Irritable, easily angered	0	1902 (40.9%)	1222 (34.4%)	1797 (50.4%)	4921 (41.8%)
	1	822 (17.7%)	764 (21.5%)	617 (17.3%)	2203 (18.7%)
	2	1004 (21.6%)	913 (25.7%)	654 (18.4%)	2571 (21.9%)
	3	593 (12.8%)	504 (14.2%)	364 (10.2%)	1461 (12.4%)
	4	325 (7.0%)	146 (4.1%)	132 (3.7%)	603 (5.1%)
Feeling Depressed or Tearful	0	2050 (44.1%)	1593 (44.9%)	2161 (60.6%)	5804 (49.4%)
	1	703 (15.1%)	677 (19.1%)	519 (14.6%)	1899 (16.1%)
	2	846 (18.2%)	706 (19.9%)	460 (12.9%)	2012 (17.1%)
	3	620 (13.3%)	397 (11.2%)	312 (8.8%)	1329 (11.3%)
	4	427 (9.2%)	176 (5.0%)	112 (3.1%)	715 (6.1%)
Feeling Frustrated or Impatient	0	1825 (39.3%)	1483 (41.8%)	1838 (51.6%)	5146 (43.8%)
	1	817 (17.6%)	721 (20.3%)	587 (16.5%)	2125 (18.1%)
	2	1018 (21.9%)	791 (22.3%)	640 (18.0%)	2449 (20.8%)
	3	606 (13.0%)	423 (11.9%)	375 (10.5%)	1404 (11.9%)
	4	380 (8.2%)	131 (3.7%)	124 (3.5%)	635 (5.4%)
Forgetfulness, poor memory	0	2302 (49.5%)	1810 (51.0%)	2074 (58.2%)	6186 (52.6%)
	1	893 (19.2%)	742 (20.9%)	518 (14.5%)	2153 (18.3%)
	2	813 (17.5%)	688 (19.4%)	636 (17.8%)	2137 (18.2%)
	3	432 (9.3%)	249 (7.0%)	245 (6.9%)	926 (7.9%)
	4	206 (4.4%)	60 (1.7%)	91 (2.6%)	357 (3.0%)
Poor Concentration	0	2248 (48.4%)	1656 (46.7%)	2004 (56.2%)	5908 (50.2%)
	1	888 (19.1%)	823 (23.2%)	561 (15.7%)	2272 (19.3%)
	2	866 (18.6%)	761 (21.4%)	605 (17.0%)	2232 (19.0%)
	3	425 (9.1%)	248 (7.0%)	286 (8.0%)	959 (8.2%)
	4	219 (4.7%)	61 (1.7%)	108 (3.0%)	388 (3.3%)
Taking Longer to Think	0	2348 (50.5%)	2042 (57.5%)	2040 (57.2%)	6430 (54.7%)
	1	864 (18.6%)	711 (20.0%)	537 (15.1%)	2112 (18.0%)
	2	831 (17.9%)	571 (16.1%)	607 (17.0%)	2009 (17.1%)
	3	414 (8.9%)	177 (5.0%)	293 (8.2%)	884 (7.5%)
	4	189 (4.1%)	48 (1.4%)	87 (2.4%)	324 (2.8%)
Blurred Vision	0	2948 (63.5%)	2149 (60.6%)	2444 (68.6%)	7541 (64.1%)
	1	759 (16.3%)	670 (18.9%)	466 (13.1%)	1895 (16.1%)
	2	587 (12.6%)	518 (14.6%)	418 (11.7%)	1523 (13.0%)
	3	249 (5.4%)	175 (4.9%)	166 (4.7%)	590 (5.0%)
	4	103 (2.2%)	37 (1.0%)	70 (2.0%)	210 (1.8%)
Light Sensitivity, easily upset by bright light	0	3110 (66.9%)	2088 (58.8%)	2484 (69.7%)	7682 (65.3%)
	1	664 (14.3%)	632 (17.8%)	476 (13.4%)	1772 (15.1%)
	2	525 (11.3%)	556 (15.7%)	356 (10.0%)	1437 (12.2%)
	3	227 (4.9%)	215 (6.1%)	176 (4.9%)	618 (5.3%)
	4	120 (2.6%)	58 (1.6%)	72 (2.0%)	250 (2.1%)
Double Vision	0	3559 (76.6%)	2688 (75.7%)	2806 (78.7%)	9053 (77.0%)
	1	592 (12.7%)	472 (13.3%)	394 (11.1%)	1458 (12.4%)
	2	310 (6.7%)	268 (7.6%)	218 (6.1%)	796 (6.8%)
	3	121 (2.6%)	96 (2.7%)	88 (2.5%)	305 (2.6%)
	4	64 (1.4%)	25 (0.7%)	58 (1.6%)	147 (1.3%)
Restlessness	0	2337 (50.3%)	1782 (50.2%)	2077 (58.3%)	6196 (52.7%)
	1	714 (15.4%)	739 (20.8%)	493 (13.8%)	1946 (16.5%)
	2	917 (19.7%)	743 (20.9%)	628 (17.6%)	2288 (19.5%)
	3	439 (9.4%)	223 (6.3%)	263 (7.4%)	925 (7.9%)
	4	239 (5.1%)	62 (1.7%)	103 (2.9%)	404 (3.4%)

Note: N: absolute frequencies, %: relative frequencies, 0: not experienced at all, 1: no more of a problem (than before), 2: a mild problem, 3: a moderate problem, 4: a severe problem.

**Table A2:** Item characteristics.

Sample	Item	*N*	*M*	*SD*	*SK*	*KU*
Dutch	Headaches	3564	0.96	1.21	**0.88**	**−0.50**
	Feeling of Dizziness	3564	0.63	1.02	**1.46**	**1.09**
	Nausea and/or Vomiting	3564	0.43	0.88	2.15	3.98
	Noise Sensitivity, easily upset by loud noise	3564	0.63	1.07	**1.56**	**1.31**
	Sleep Disturbance	3564	1.19	1.33	**0.68**	**−0.82**
	Fatigue, tiring more easily	3564	1.44	1.38	**0.39**	**−1.17**
	Being Irritable, easily angered	3564	0.99	1.20	**0.89**	**−0.38**
	Feeling Depressed or Tearful	3564	0.79	1.15	**1.25**	**0.37**
	Feeling Frustrated or Impatient	3564	0.98	1.20	**0.90**	**−0.39**
	Forgetfulness, poor memory	3564	0.81	1.11	**1.13**	**0.19**
	Poor Concentration	3564	0.86	1.14	**1.09**	**0.07**
	Taking Longer to Think	3564	0.84	1.12	**1.08**	**0.03**
	Blurred Vision	3564	0.58	1.00	**1.66**	**1.89**
	Light Sensitivity, easily upset by bright light	3564	0.56	0.99	**1.76**	2.22
	Double Vision	3564	0.37	0.84	2.52	6.11
	Restlessness	3564	0.83	1.13	**1.12**	**0.14**
English	Headaches	4646	1.11	1.19	**0.65**	**−0.73**
	Feeling of Dizziness	4646	0.81	1.10	**1.10**	**0.10**
	Nausea and/or Vomiting	4646	0.55	0.94	**1.76**	2.45
	Noise Sensitivity, easily upset by loud noise	4646	0.70	1.11	**1.45**	**1.02**
	Sleep Disturbance	4646	1.42	1.38	**0.44**	**−1.11**
	Fatigue, tiring more easily	4646	1.59	1.38	**0.27**	**−1.20**
	Being Irritable, easily angered	4646	1.27	1.30	**0.60**	**−0.85**
	Feeling Depressed or Tearful	4646	1.28	1.38	**0.63**	**−0.96**
	Feeling Frustrated or Impatient	4646	1.33	1.33	**0.55**	**−0.92**
	Forgetfulness, poor memory	4646	1.00	1.20	**0.94**	**−0.23**
	Poor Concentration	4646	1.03	1.21	**0.90**	**−0.29**
	Taking Longer to Think	4646	0.97	1.19	**0.96**	**−0.19**
	Blurred Vision	4646	0.67	1.03	**1.48**	**1.30**
	Light Sensitivity, easily upset by bright light	4646	0.62	1.03	**1.64**	**1.81**
	Double Vision	4646	0.39	0.83	2.36	5.33
	Restlessness	4646	1.04	1.24	**0.87**	**−0.42**
Italian	Headaches	3549	1.19	1.13	**0.38**	**−1.07**
	Feeling of Dizziness	3549	0.69	1.01	**1.28**	**0.61**
	Nausea and/or Vomiting	3549	0.62	0.90	**1.36**	**1.05**
	Noise Sensitivity, easily upset by loud noise	3549	0.75	1.06	**1.23**	**0.45**
	Sleep Disturbance	3549	1.14	1.22	**0.65**	**−0.75**
	Fatigue, tiring more easily	3549	1.41	1.23	**0.31**	**−1.05**
	Being Irritable, easily angered	3549	1.32	1.20	**0.43**	**−0.89**
	Feeling Depressed or Tearful	3549	1.12	1.24	**0.75**	**−0.59**
	Feeling Frustrated or Impatient	3549	1.15	1.19	**0.64**	**−0.71**
	Forgetfulness, poor memory	3549	0.87	1.06	**0.95**	**−0.09**
	Poor Concentration	3549	0.94	1.05	**0.83**	**−0.25**
	Taking Longer to Think	3549	0.73	0.99	**1.21**	**0.60**
	Blurred Vision	3549	0.67	0.97	**1.30**	**0.80**
	Light Sensitivity, easily upset by bright light	3549	0.74	1.03	**1.22**	**0.52**
	Double Vision	3549	0.39	0.80	2.18	4.35
	Restlessness	3549	0.89	1.05	**0.91**	**−0.12**
Total (characteristics across language samples)	*Min*	3549.00	0.37	0.80	0.27	−1.20
	*Max*	4646.00	1.59	1.38	2.52	6.11
	*M*	3919.67	0.90	1.12	1.10	0.46
	*SD*	524.03	0.32	0.16	0.56	1.79

Note: *N*: absolute frequencies, *M*: mean, *SD*: standard deviation, *SK*: skewness, *KU*: kurtosis, *Min*: minimum, *Max*: maximum. Values in **bold** indicate acceptable *SK* and *KU* (−2 to +2).

**Table A3:** Psychometric properties of the RPQ in the general population samples.

Sample	Item	Cronbach’s Alpha	Alpha If Item Omitted	Split-Half Reliability	Item-Total Correlation
Dutch	Headaches	**0.94**	**0.94**	**0.96**	**0.55**
	Feeling of Dizziness		**0.94**		**0.62**
	Nausea and/or Vomiting		**0.94**		**0.56**
	Noise Sensitivity, easily upset by loud noise		**0.94**		**0.64**
	Sleep Disturbance		**0.94**		**0.65**
	Fatigue, tiring more easily		**0.93**		**0.71**
	Being Irritable, easily angered		**0.93**		**0.73**
	Feeling Depressed or Tearful		**0.93**		**0.73**
	Feeling Frustrated or Impatient		**0.93**		**0.74**
	Forgetfulness, poor memory		**0.93**		**0.74**
	Poor Concentration		**0.93**		**0.78**
	Taking Longer To Think		**0.93**		**0.76**
	Blurred Vision		**0.93**		**0.65**
	Light Sensitivity, easily upset by bright light		**0.93**		**0.66**
	Double Vision		**0.94**		**0.59**
	Restlessness		**0.93**		**0.73**
English	Headaches	**0.95**	**0.94**	**0.96**	**0.57**
	Feeling of Dizziness		**0.94**		**0.67**
	Nausea and/or Vomiting		**0.94**		**0.60**
	Noise Sensitivity, easily upset by loud noise		**0.94**		**0.68**
	Sleep Disturbance		**0.94**		**0.68**
	Fatigue, tiring more easily		**0.94**		**0.73**
	Being Irritable, easily angered		**0.94**		**0.76**
	Feeling Depressed or Tearful		**0.94**		**0.75**
	Feeling Frustrated or Impatient		**0.94**		**0.77**
	Forgetfulness, poor memory		**0.94**		**0.76**
	Poor Concentration		**0.94**		**0.80**
	Taking Longer to Think		**0.94**		**0.79**
	Blurred Vision		**0.94**		**0.65**
	Light Sensitivity, easily upset by bright light		**0.94**		**0.67**
	Double Vision		**0.94**		**0.59**
	Restlessness		**0.94**		**0.75**
Italian	Headaches	**0.92**	**0.92**	**0.95**	**0.49**
	Feeling of Dizziness		**0.92**		**0.56**
	Nausea and/or Vomiting		**0.92**		**0.55**
	Noise Sensitivity, easily upset by loud noise		**0.92**		**0.60**
	Sleep Disturbance		**0.92**		**0.63**
	Fatigue, tiring more easily		**0.92**		**0.70**
	Being Irritable, easily angered		**0.92**		**0.68**
	Feeling Depressed or Tearful		**0.92**		**0.68**
	Feeling Frustrated or Impatient		**0.92**		**0.72**
	Forgetfulness, poor memory		**0.92**		**0.67**
	Poor Concentration		**0.92**		**0.72**
	Taking Longer to Think		**0.92**		**0.70**
	Blurred Vision		**0.92**		**0.60**
	Light Sensitivity, easily upset by bright light		**0.92**		**0.60**
	Double Vision		**0.92**		**0.54**
	Restlessness		**0.92**		**0.68**
Total (characteristics across language samples)	*Min*	0.92	0.92	0.95	0.49
	*Max*	0.95	0.94	0.96	0.80
	*M*	0.93	0.93	0.95	0.67
	*SD*	0.01	0.01	0.01	0.08

Note: *Min*: minimum, *Max*: maximum, *M*: mean, *SD*: standard deviation. Values in **bold** are within acceptable ranges.

**Table A4:** Examination of the factorial structure of the RPQ in the general population samples.

Model Fit Indices	Original One-Factor Model			Three-Factor model Smith-Seemiller et al. (2003) [21]		
	Dutch	English	Italian	Dutch	English	Italian
χ2	7042.459	5050.506	4054.506	3382.947	2056.336	1846.032
df	104	104	104	101	101	101
*p*	<0.001	<0.001	<0.001	<0.001	<0.001	<0.001
χ2/df	68	49	39	33	20	18
CFI	**0.988**	**0.979**	**0.989**	**0.994**	**0.992**	**0.995**
TLI	**0.987**	**0.976**	**0.987**	**0.993**	**0.990**	**0.994**
RMSEA	0.120	0.116	0.103	**0.084**	**0.074**	**0.070**
CI_90%_	[0.117, 0.122]	[0.113, 0.119]	[0.101, 0.106]	**[0.081, 0.086]**	**[0.071, 0.077]**	**[0.067, 0.072]**
SRMR	0.075	0.080	0.069	**0.061**	**0.058**	**0.053**

Note. χ2: chi square, df: degrees of freedom, χ2/df: ratio (cut-off: ≤ 2), *p*: *p*-value, CFI: Comparative Fit Index (cut-off: >0.95), TLI: Tucker–Lewis Index (cut-off: >0.95), RMSEA: root mean square error of approximation (cut-off: 0.05–0.10) with 90% confidence interval (CI), SRMR: standardized root mean square residual (cut-off: <0.08). Values in **bold** are within acceptable range. The grey-shaded model (i.e., the three-factors model) provides a better model fit.

**Table A5:** Results of the measurement invariance (MI) analyses.

Samples	Constrains	χ^2^	df	*p*	CFI	TLI	RMSEA	CI_90%_	Δχ^2^	Δdf	Δp	ΔCFI	ΔRMSEA
Dutch (General population sample vs. TBI sample)	baseline	3604.965	202	<0.001	**0.971**	**0.966**	**0.090**	**[0.088, 0.093]**	-	-	-	-	-
	thresholds	3840.568	233	<0.001	**0.969**	**0.968**	**0.086**	**[0.084, 0.089]**	155.04	31	<0.001	**0.002**	**0.004**
	intercepts and thresholds	3665.442	246	<0.001	**0.971**	**0.972**	**0.082**	**[0.080, 0.084]**	15.154	13	**0.298**	**−0.002**	**0.004**
English (General population sample vs. TBI sample)	baseline	5020.305	202	<0.001	**0.974**	**0.969**	**0.099**	**[0.097, 0.101]**	-	-	-	-	-
	thresholds	5093.583	233	<0.001	**0.973**	**0.973**	**0.093**	**[0.090, 0.095]**	26.53	31	**0.695**	**0.001**	**0.006**
	intercepts and thresholds	4713.479	246	<0.001	**0.976**	**0.976**	**0.086**	**[0.084, 0.089]**	14.13	13	**0.365**	**−0.003**	**0.007**
Italian (General population sample vs. TBI sample)	baseline	3413.452	202	<0.001	**0.961**	**0.954**	**0.091**	**[0.089, 0.094]**	-	-	-	-	-
	thresholds	3521.641	233	<0.001	**0.960**	**0.959**	**0.086**	**[0.084, 0.089]**	77.73	31	<0.001	**0.001**	**0.005**
	intercepts and thresholds	3246.144	246	<0.001	**0.964**	**0.964**	**0.080**	**[0.078, 0.082]**	13.82	13	**0.387**	**−0.004**	**0.006**
Dutch vs. English vs. Italian (General population samples)	baseline	12,665.04	303	<0.001	**0.963**	**0.956**	**0.102**	**[0.101, 0.104]**	-	-	-	-	-
	thresholds	13,347.41	365	<0.001	**0.961**	**0.962**	**0.095**	**[0.094, 0.097]**	145.61	62	<0.001	**0.002**	**0.007**
	intercepts and thresholds	12,725.06	391	<0.001	**0.963**	**0.966**	**0.090**	**[0.088, 0.091]**	94.79	26	<0.001	**−0.002**	**0.005**

Note. χ^2^: chi square, df: degree of freedom, χ^2^/df: ratio (cut-off: ≤2), *p*: *p*-value, CFI: Comparative Fit Index (cut-off: >0.95), TLI: Tucker–Lewis Index (cut-off: >0.95), RMSEA: root mean square error of approximation (cut-off: <0.08) with 90% confidence interval (CI), Δχ^2^: change in chi square values between compared models, Δdf: change in degrees of freedom between compared models, ΔCFI: change in CFI between compared models (cut-off: <0.01), ΔRMSEA: change in RMSEA between compared models (cut-off: ≤0.01). Values in **bold** indicate at least satisfactory/mediocre model fit according to the respective cut-offs and/or are within acceptable range.

**Table A6:** Results of regression analyses.

		RPQ Total Score				Somatic				Emotional				Cognitive			
Variable	Reference Group	Estimate	S.E.	*t*	*p*	Estimate	S.E.	*t*	*p*	Estimate	S.E.	*t*	*p*	Estimate	S.E.	*t*	*p*
(Intercept)	-	8.76	0.67	13.00	0.000	4.34	0.36	12.12	**<0.001**	2.85	0.23	12.19	**<0.001**	1.56	0.18	8.94	**<0.001**
Italian	English	−1.58	0.72	−2.20	**0.028**	−0.49	0.38	−1.29	0.197	−0.72	0.25	−2.89	**0.004**	−0.37	0.19	−1.97	**0.048**
Dutch		−0.34	0.74	−0.46	0.647	0.13	0.39	0.33	0.739	−0.65	0.26	−2.54	**0.011**	0.18	0.19	0.95	0.342
41–64 years	18–40 years	−2.80	0.67	−4.18	**<0.001**	−1.48	0.36	−4.17	**<0.001**	−0.74	0.23	−3.20	**0.001**	−0.57	0.17	−3.28	**0.001**
65–75 years		−5.41	0.94	−5.74	**<0.001**	−2.51	0.50	−4.99	**<0.001**	−1.87	0.33	−5.71	**<0.001**	−1.04	0.24	−4.23	**<0.001**
Female	Male	2.50	0.63	3.94	**<0.001**	1.25	0.34	3.71	**<0.001**	0.87	0.22	3.93	**<0.001**	0.39	0.16	2.34	**0.019**
Middle	Low	−1.95	0.67	−2.90	**0.004**	−0.97	0.36	−2.71	**0.007**	−0.51	0.23	−2.20	**0.028**	−0.46	0.17	−2.66	**0.008**
High		−2.37	0.74	−3.22	**0.001**	−1.13	0.39	−2.87	**0.004**	−0.82	0.26	−3.19	**0.001**	−0.43	0.19	−2.24	**0.025**
At least one health complaint	No health complaints	13.17	0.64	20.47	**<0.001**	6.10	0.34	17.81	**<0.001**	4.30	0.22	19.22	**<0.001**	2.78	0.17	16.63	**<0.001**
Italian * 41–64 years	English * 18–40 years	2.02	0.58	3.48	**0.001**	1.05	0.31	3.41	**0.001**	0.56	0.20	2.77	**0.006**	0.41	0.15	2.71	**0.007**
Dutch * 41–64 years		−0.51	0.56	−0.91	0.362	−0.58	0.30	−1.94	0.053	0.12	0.19	0.64	0.522	−0.06	0.15	−0.40	0.687
Italian * 65–75 years	English * 18–40 years	3.76	0.87	4.31	**<0.001**	1.57	0.46	3.39	**0.001**	1.05	0.30	3.45	**0.001**	1.14	0.23	5.05	**<0.001**
Dutch * 65–75 years		0.69	0.87	0.79	0.428	−0.01	0.47	−0.03	0.975	0.49	0.30	1.61	0.108	0.22	0.23	0.97	0.332
Italian * Female	English * Male	1.13	0.53	2.11	**0.035**	0.50	0.28	1.76	0.078	0.58	0.19	3.13	**0.002**	0.04	0.14	0.31	0.754
Dutch * Female		−0.90	0.52	−1.72	0.085	−0.56	0.28	−2.02	**0.043**	−0.15	0.18	−0.82	0.413	−0.19	0.14	−1.39	0.164
Italian * Middle	English * Low	0.64	0.62	1.03	0.305	0.07	0.33	0.21	0.833	0.41	0.22	1.92	0.055	0.15	0.16	0.95	0.341
Dutch * Middle		0.27	0.65	0.42	0.677	0.20	0.34	0.59	0.555	0.15	0.22	0.68	0.498	−0.09	0.17	−0.51	0.612
Italian * High		2.57	0.84	3.06	**0.002**	1.26	0.45	2.83	**0.005**	0.88	0.29	3.04	**0.002**	0.42	0.22	1.93	**0.054**
Dutch * High		−0.19	0.72	−0.26	0.794	0.08	0.38	0.22	0.826	0.12	0.25	0.47	0.638	−0.39	0.19	−2.09	0.037
Italian * At least one health complaint	English * No health complaints	−4.15	0.54	−7.74	**<0.001**	−1.62	0.29	−5.69	**<0.001**	−1.44	0.19	−7.76	**<0.001**	−1.08	0.14	−7.76	**<0.001**
Dutch * At least one health complaint		−3.13	0.53	−5.95	**<0.001**	−0.96	0.28	−3.44	**0.001**	−1.50	0.18	−8.24	**<0.001**	−0.66	0.14	−4.86	**<0.001**
41–64 years * Female	18–40 years * Male	−0.07	0.47	−0.15	0.878	0.34	0.25	1.36	0.173	−0.38	0.16	−2.32	**0.020**	−0.03	0.12	−0.28	**0.784**
65–75 years * Female		−0.59	0.71	−0.83	0.408	0.02	0.38	0.05	0.959	−0.39	0.25	−1.60	0.110	−0.21	0.18	−1.16	0.247
41–64 * Middle	18–40 years * Low	0.60	0.57	1.05	0.292	0.36	0.30	1.20	0.230	0.11	0.20	0.54	0.588	0.13	0.15	0.88	0.381
65–75 * Middle		0.33	0.84	0.39	0.697	0.31	0.44	0.70	0.482	0.03	0.29	0.09	0.929	−0.01	0.22	−0.06	0.950
41–64 * High		0.64	0.67	0.96	0.338	0.39	0.36	1.10	0.271	0.06	0.23	0.27	0.790	0.19	0.17	1.08	0.279
65–75 * High		0.57	1.01	0.57	0.572	0.12	0.54	0.22	0.823	0.50	0.35	1.41	0.159	−0.04	0.26	−0.16	0.870
41–64*At least one health complaint	18–40 * No health complaints	−0.66	0.47	−1.41	0.157	−0.29	0.25	−1.16	0.246	−0.37	0.16	−2.30	**0.021**	0.00	0.12	0.01	0.993
65–75 * At least one health complaint		−4.39	0.71	−6.17	**<0.001**	−1.97	0.38	−5.20	**<0.001**	−1.62	0.25	−6.57	**<0.001**	−0.80	0.18	−4.32	**<0.001**
Female * Middle	Male * Low	−0.34	0.51	−0.67	0.504	−0.14	0.27	−0.50	0.614	−0.24	0.18	−1.33	0.183	0.03	0.13	0.24	0.808
Female * High		−0.52	0.62	−0.84	0.399	−0.15	0.33	−0.47	0.638	−0.20	0.21	−0.92	0.356	−0.17	0.16	−1.05	0.294
Female * At least one health complaint	Male * No health complaints	0.52	0.43	1.21	0.225	0.34	0.23	1.49	0.136	0.18	0.15	1.23	0.220	0.00	0.11	−0.01	0.989
Middle * At least one health complaint	Low * No health complaints	0.53	0.52	1.02	0.308	0.22	0.28	0.80	0.422	0.28	0.18	1.53	0.127	0.03	0.14	0.24	0.809
High * At least one health complaint		−0.80	0.62	−1.29	0.196	−0.35	0.33	−1.05	0.294	−0.18	0.22	−0.85	0.395	−0.27	0.16	−1.70	0.090

Note: *: interaction between the variables; Estimate: regression coefficient; S.E.: standard error; *t*: *t*-value; *p*: *p*-value; values in **bold** are significant at 5%.

## Appendix B

**Table A7:** Reference values for the RPQ scale scores obtained from the English general population sample stratified by sex, health status, and age.

	Gender × Health Status x Age				Low Symptom Severity		−1 SD			Md			+1 SD		High Symptom Severity
Scale	Gender	Health status	Age	N	2.5%	5%	16%	30%	40%	50%	60%	70%	85%	95%	97.5%
Somatic	Male	Healthy	18–40	566	0	0	0	0	0	2	2	4	8	16	18
			41–64	460	0	0	0	0	0	0	2	2	4	10	14
			65–75	118	0	0	0	0	0	0	0	2	5	12	12
		At least one chronic health condition	18–40	446	0	0	2	4	6	8	10	13	18	24	28
			41–64	542	0	0	0	4	5	6	8	10	16	24	27
			65–75	156	0	0	0	0	2	4	5	7	11	15	18
	Female	Healthy	18–40	474	0	0	0	0	2	3	4	6	10	17	19
			41–64	422	0	0	0	0	0	2	3	4	8	13	17
			65–75	119	0	0	0	0	0	2	2	4	6	11	13
		At least one chronic health condition	18–40	564	0	0	3	6	8	9	11	14	19	26	28
			41–64	602	0	0	3	6	7	9	11	14	19	25	27
			65–75	177	0	0	0	3	4	6	7	9	13	18	21
			Total	4646	0	0	0	2	2	4	6	9	14	21	25
Emotional	Male	Healthy	18–40	566	0	0	0	0	0	0	2	3	7	10	12
			41–64	460	0	0	0	0	0	0	0	2	4	8	9
			65–75	118	0	0	0	0	0	0	0	0	4	8	9
		At least one chronic health condition	18–40	446	0	0	0	2	5	7	8	10	12	15	16
			41–64	542	0	0	0	2	4	5	7	9	12	15	16
			65–75	156	0	0	0	0	0	2	3	4	8	12	14
	Female	Healthy	18–40	474	0	0	0	0	0	0	2	4	8	11	12
			41–64	422	0	0	0	0	0	0	0	2	6	9	10
			65–75	119	0	0	0	0	0	0	0	0	2	7	9
		At least one chronic health condition	18–40	564	0	0	2	4	6	8	9	11	13	16	16
			41–64	602	0	0	0	2	4	6	8	10	12	15	16
			65–75	177	0	0	0	0	0	2	4	6	9	11	12
			Total	4646	0	0	0	0	0	2	4	7	10	14	15
Cognitive	Male	Healthy	18–40	566	0	0	0	0	0	0	0	0	4	7	9
			41–64	460	0	0	0	0	0	0	0	0	2	6	6
			65–75	118	0	0	0	0	0	0	0	0	2	6	6
		At least one chronic health condition	18–40	446	0	0	0	0	2	3	5	6	9	11	12
			41–64	542	0	0	0	0	0	2	4	6	8	11	12
			65–75	156	0	0	0	0	0	0	2	2	6	9	9
	Female	Healthy	18–40	474	0	0	0	0	0	0	0	0	4	7	9
			41–64	422	0	0	0	0	0	0	0	0	2	6	8
			65–75	119	0	0	0	0	0	0	0	0	1	4	4
		At least one chronic health condition	18–40	564	0	0	0	0	2	4	6	6	9	12	12
			41–64	602	0	0	0	0	2	4	6	6	9	12	12
			65–75	177	0	0	0	0	0	0	0	2	6	8	9
			Total	4646	0	0	0	0	0	0	2	4	7	9	11

Note: 50% percentiles represent 50% of the distribution corresponding to the median (Md); SD: standard deviation; values from −1 standard deviation (16%) to +1 standard deviation (85%) are within the normal range (i.e., not clinically relevant symptom severity); values below 16% indicate low symptoms severity (i.e., absence of RPQ symptoms) and values above 85% indicate high symptom severity (i.e., presence of clinically relevant RPQ symptoms).

**Table A8:** Reference values for the RPQ scale scores obtained from the Italian general population sample stratified by sex, health status, and age.

	Gender × Health Status × Age				Low Symptom Severity		−1 SD			Md			+1 SD		High Symptom Severity
Scale	Gender	Health status	Age	N	2.5%	5%	16%	30%	40%	50%	60%	70%	85%	95%	97.5%
Somatic	Male	Healthy	18–40	467	0	0	0	0	0	2	2	4	7	14	18
			41–64	454	0	0	0	0	0	2	2	4	8	14	18
			65–75	106	0	0	0	0	0	2	2	4	7	9	10
		At least one chronic health condition	18–40	227	0	0	2	4	5	6	9	12	16	19	23
			41–64	391	0	0	0	2	4	6	7	10	14	21	24
			65–75	125	0	0	0	2	2	4	6	8	10	17	19
	Female	Healthy	18–40	419	0	0	0	2	3	4	5	6	10	16	18
			41–64	403	0	0	0	2	2	4	5	7	11	16	18
			65–75	91	0	0	0	0	0	0	2	4	7	13	14
		At least one chronic health condition	18–40	302	0	0	2	6	7	9	10	13	18	23	25
			41–64	435	0	0	2	5	7	9	11	13	18	23	26
			65–75	129	0	0	2	4	4	6	8	10	15	22	25
			Total	3549	0	0	0	2	2	4	6	8	13	19	22
Emotional	Male	Healthy	18–40	467	0	0	0	0	0	0	2	2	6	9	10
			41–64	454	0	0	0	0	0	0	0	2	6	9	11
			65–75	106	0	0	0	0	0	0	0	0	3	6	7
		At least one chronic health condition	18–40	227	0	0	0	2	4	5	6	7	10	13	15
			41–64	391	0	0	0	0	2	4	5	7	10	12	14
			65–75	125	0	0	0	0	0	2	2	2	6	11	13
	Female	Healthy	18–40	419	0	0	0	0	0	2	4	6	8	11	11
			41–64	403	0	0	0	0	0	0	2	4	8	11	12
			65–75	91	0	0	0	0	0	0	0	0	6	9	10
		At least one chronic health condition	18–40	302	0	0	0	3	6	7	8	9	12	14	16
			41–64	435	0	0	0	2	4	6	7	9	11	13	15
			65–75	129	0	0	0	0	2	4	5	7	9	12	13
			Total	3549	0	0	0	0	0	2	4	6	9	12	13
Cognitive	Male	Healthy	18–40	467	0	0	0	0	0	0	0	0	2	6	7
			41–64	454	0	0	0	0	0	0	0	0	2	6	7
			65–75	106	0	0	0	0	0	0	0	0	2	4	5
		At least one chronic health condition	18–40	227	0	0	0	0	0	2	3	5	7	9	9
			41–64	391	0	0	0	0	0	0	2	4	6	9	10
			65–75	125	0	0	0	0	0	0	2	4	6	9	9
	Female	Healthy	18–40	419	0	0	0	0	0	0	0	2	4	7	9
			41–64	403	0	0	0	0	0	0	0	0	6	7	9
			65–75	91	0	0	0	0	0	0	0	0	4	6	8
		At least one chronic health condition	18–40	302	0	0	0	0	0	2	4	5	7	9	12
			41–64	435	0	0	0	0	0	2	3	6	7	9	9
			65–75	129	0	0	0	0	0	0	2	4	6	9	9
			Total	3549	0	0	0	0	0	0	0	2	6	8	9

Note. 50% percentiles represent 50% of the distribution corresponding to the median (Md); SD: standard deviation; values from −1 standard deviation (16%) to +1 standard deviation (85%) are within the normal range (i.e., not clinically relevant symptom severity); values below 16% indicate low symptoms severity (i.e., absence of RPQ symptoms) and values above 85% indicate high symptom severity (i.e., presence of clinically relevant RPQ symptoms).

**Table A9:** Reference values for the RPQ scale scores obtained from the Dutch general population sample stratified by sex, health status, and age.

	Gender × Health Status × Age				Low Symptom Severity		−1 SD			Md			+1 SD		High Symptom Severity
Scale	Gender	Health status	Age	N	2.5%	5%	16%	30%	40%	50%	60%	70%	85%	95%	97.5%
Somatic	Male	Healthy	18–40	444	0	0	0	0	0	2	2	4	7	16	19
			41–64	412	0	0	0	0	0	0	2	2	6	12	15
			65–75	94	0	0	0	0	0	0	0	2	2	4	7
		At least one chronic health condition	18–40	276	0	0	0	3	5	8	10	14	18	23	28
			41–64	447	0	0	0	2	4	5	7	9	15	23	26
			65–75	109	0	0	0	0	0	2	4	6	10	19	23
	Female	Healthy	18–40	363	0	0	0	0	2	2	4	6	10	14	16
			41–64	298	0	0	0	0	0	2	2	4	7	13	15
			65–75	66	0	0	0	0	0	2	2	2	5	8	10
		At least one chronic health condition	18–40	372	0	0	3	6	7	9	11	13	18	23	27
			41–64	536	0	0	2	4	5	6	8	10	15	20	23
			65–75	147	0	0	0	2	4	6	7	9	12	20	24
			Total	3564	0	0	0	0	2	4	5	8	13	19	23
Emotional	Male	Healthy	18–40	444	0	0	0	0	0	0	0	2	4	9	10
			41–64	412	0	0	0	0	0	0	0	0	4	8	10
			65–75	94	0	0	0	0	0	0	0	0	0	2	5
		At least one chronic health condition	18–40	276	0	0	0	0	2	4	5	7	10	13	15
			41–64	447	0	0	0	0	0	2	4	6	10	12	14
			65–75	109	0	0	0	0	0	0	0	2	5	9	11
	Female	Healthy	18–40	363	0	0	0	0	0	0	2	3	6	10	11
			41–64	298	0	0	0	0	0	0	0	2	4	9	10
			65–75	66	0	0	0	0	0	0	0	0	2	6	7
		At least one chronic health condition	18–40	372	0	0	0	2	4	5	7	8	11	14	16
			41–64	536	0	0	0	0	0	2	4	6	10	12	14
			65–75	147	0	0	0	0	0	0	2	4	6	10	11
			Total	3564	0	0	0	0	0	0	2	4	8	12	13
Cognitive	Male	Healthy	18–40	444	0	0	0	0	0	0	0	0	4	6	8
			41–64	412	0	0	0	0	0	0	0	0	2	6	6
			65–75	94	0	0	0	0	0	0	0	0	0	3	6
		At least one chronic health condition	18–40	276	0	0	0	0	0	2	4	5	7	10	12
			41–64	447	0	0	0	0	0	0	3	6	7	10	11
			65–75	109	0	0	0	0	0	0	0	2	6	7	8
	Female	Healthy	18–40	363	0	0	0	0	0	0	0	2	4	7	8
			41–64	298	0	0	0	0	0	0	0	0	2	6	7
			65–75	66	0	0	0	0	0	0	0	0	2	6	6
		At least one chronic health condition	18–40	372	0	0	0	0	0	3	4	6	8	11	12
			41–64	536	0	0	0	0	0	0	4	5	7	9	10
			65–75	147	0	0	0	0	0	0	2	2	6	9	9
			Total	3564	0	0	0	0	0	0	0	2	6	9	10

Note: 50% percentiles represent 50% of the distribution corresponding to the median (Md); SD: standard deviation; values from −1 standard deviation (16%) to +1 standard deviation (85%) are within the normal range (i.e., not clinically relevant symptom severity); values below 16% indicate low symptoms severity (i.e., absence of RPQ symptoms) and values above 85% indicate high symptom severity (i.e., presence of clinically relevant RPQ symptoms).
